# Proteomic characterization of the *Rph15* barley resistance gene-mediated defence responses to leaf rust

**DOI:** 10.1186/1471-2164-13-642

**Published:** 2012-11-20

**Authors:** Letizia Bernardo, Bhakti Prinsi, Alfredo Simone Negri, Luigi Cattivelli, Luca Espen, Giampiero Valè

**Affiliations:** 1 CRA-Consiglio per la ricerca e la sperimentazione in agricoltura, Genomics Research Centre, Via S. Protaso 302, Fiorenzuola d’Arda, PC, I-29017, Italy; 2 Dipartimento di Scienze agrarie ambientali – Produzione – Territorio – Agroenergia (Di.S.A.A), University of Milan, via Celoria 2, Milano, I-20133, Italy; 3 CRA-Consiglio per la ricerca e la sperimentazione in agricoltura, Rice Research Unit, S.S. 11 to Torino, Km 2,5, Vercelli, 13100, Italy

**Keywords:** Barley, Leaf rust, Resistance gene, Rph15, Proteomics, Near isogenic lines

## Abstract

**Background:**

Leaf rust, caused by the biotrophic fungal pathogen *Puccinia hordei*, is one of the most important foliar disease of barley (*Hordeum vulgare*) and represents a serious threat in many production regions of the world. The leaf rust resistance gene *Rph15* is of outstanding interest for resistance breeding because it confers resistance to over 350 *Puccinia hordei* isolates collected from around the world. Molecular and biochemical mechanisms responsible for the *Rph15* effectiveness are currently not investigated. The aim of the present work was to study the *Rph15*-based defence responses using a proteomic approach.

**Results:**

Protein pattern changes in response to the leaf rust pathogen infection were investigated in two barley near isogenic lines (NILs), Bowman (leaf rust susceptible) and Bowman-*Rph15* (leaf rust resistant), differing for the introgression of the leaf rust resistance gene *Rph15*. Two infection time points, 24 hours and four days post inoculation (dpi), were analysed. No statistically significant differences were identified at the early time point, while at 4 dpi eighteen protein spots were significantly up or down regulated with a fold-change equal or higher than two in response to pathogen infection. Almost all the pathogen-responsive proteins were identified in the Bowman-*Rph15* resistant NIL. Protein spots were characterized by LC-MS/MS analysis and found to be involved in photosynthesis and energy metabolism, carbohydrate metabolism, protein degradation and defence. Proteomic data were complemented by transcriptional analysis of the respective genes. The identified proteins can be related to modulation of the photosynthetic apparatus components, re-direction of the metabolism to sustain defence responses and deployment of defence proteins.

**Conclusions:**

The identification of leaf rust infection-modulated defence responses restricted to the resistant NIL support the hypothesis that basal defence responses of Bowman, but not the *Rph15* resistance gene-based ones, are suppressed or delayed by pathogen effectors to levels below the detection power of the adopted proteomic approach. Additionally, *Rph15*-mediated resistance processes identified mainly resides on a modulation of primary metabolism, affecting photosyntesis and carbohydrate pool.

## Background

The fungus *Puccinia hordei* is a biotrophic pathogen causal agent of leaf rust, a serious leaf disease of barley worldwide. This pathogen causes serious economic losses with yield reductions by up to 62% [1,2] and adversely affects grain quality by reducing grain weight and increased levels of undesirable protein in the barley growing region of the world [3]. Rust fungi have a complex life cycle that involves two parasitic stages, dikaryotic and monokaryotic [4]. The dikaryotic stage is the form causing rust disease by attacking mesophyll tissues until pathogen injures the epidermis to release urediospores [5].

Barley resistance to leaf rust pathogens is governed by major resistance (R) genes (*Rph* genes) that are race-specific. Most *Rph* genes confer complete seedling resistance associated with necrosis (or hypersensitive response, HR) of the plant cells attacked by the pathogen sporelings [6], while some *Rph* resistance genes confer incomplete resistance, in which the fungus forms small uredinia surrounded by chlorotic or necrotic plant tissues. This second resistance type is a non-HR (non-hypersensitivity resistance) polygenically inherited leaf rust resistance, which was termed “partial resistance” and is not associated with plant cell necrosis [7]. Several seedling resistance genes were identified from cultivated and wild barley, of which 19 were designed *Rph1* to *Rph19*[8]. The resistance provided by single *Rph* genes has often been overcome by new pathotypes, believed to have arisen after gene mutations. As a direct consequence, the number of effective *Rph* genes available to breeders is decreasing rapidly, suggesting the need for a new gene deployment strategy [9]. The leaf rust resistance gene *Rph15*, located on chromosome 2HS [1], was derived from PI 355447, an accession of wild barley (*Hordeum vulgare* subsp. *spontaneum*) collected in Israel. When evaluated for its reaction toward a worldwide collection of over 350 *P. hordei* isolates, it conferred resistance to all but the isolates 90–3, from Israel. *Rph15* is one of the most broadly effective resistance genes and it is therefore useful in barley breeding programs for leaf rust resistance.

Barley interaction with the leaf rust pathogen represents a model to understand the molecular basis of both race-specific and partial resistance. Molecular basis of partial resistance were recently investigated using eQTL (expression Quantitative Trait Loci) analyses carried out in *P. hordei* infected doubled haploid lines and QTL-NILs (QTL-Near Isogenic Lines) [10,11]. These studies provided an overview of the responsive dynamic defence process and identified several candidate genes as being co-localized with the phenotypic QTL. No additional published microarray studies were dedicated to barley-*P. hordei* interaction.

In addition to transcriptional studies, proteomic techniques can provide insight into the molecular mechanisms underpinning resistance gene-based plant defence responses. The compatible interaction between wheat and the leaf rust pathogen *P. triticina* was investigated at 3, 6 and 9 dpi (days post inoculation) and only at the latter time point of inoculation seven plant proteins involved in translation and stress responses were identified as pathogen-responsive [12]. No published proteomic studies have been performed on barley leaves infected with leaf rust.

In this work, barley responses to leaf rust infection were investigated, in two barley near isogenic lines differing for the introgression of the broad effective leaf rust resistance gene *Rph15*. A proteomic study was performed at early (24 hours) and late (4 dpi) infection times and, after analysis of protein pattern changes, it was observed that only at the late time points and in the resistant NIL differential protein accumulation occurs in response to pathogen inoculation. The differentially expressed proteins were involved in photosynthesis, carbon metabolism, defence responses and secondary metabolism.

## Results and discussion

### Experimental design and 2-DE analysis

**Figure 1:**
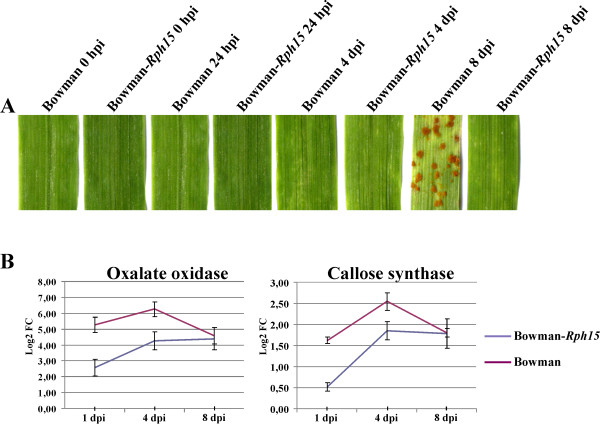
**Phenotype and defense genes activation of barley leaves subjected to proteomic analyses.** (**A**) Barley leaves images of the two NILs Bowman and Bowman-*Rph15* inoculated with the leaf rust pathogen at the indicated time points and utilized for the proteomic analyses. After sampling, in some plants for each biological replicate the disease was left to proceed until 8 dpi to assess the success of the infection experiments. (**B**) The same genotypes and time points of inoculation as in (**A**) were verified for the transcriptional activation of the defense related genes coding for oxalate oxidase and callose synthase using quantitative RT-PCR analysis. Normalization was carried out with the β-actin constitutively expressed gene. Values are expressed as log2 fold changes of transcript levels in the inoculated samples with respect to the transcript levels in un-inoculated barley leaves. Error bars represent SD across all RT-PCR replicates (three from each of two independent inoculations). Statistical significance of differential expression was evaluated with a Wilcoxon two group test (P<0.05, Methods).

In this study, the defence responses to leaf rust of two near-isogenic barley lines, Bowman and Bowman*-Rph15* differing for the presence/absence of the broad effective leaf rust resistance gene *Rph15*, were investigated using a proteomic approach. The utilization of NILs allows to relate the pathogen-responsive changes in protein accumulation observed between the two NILs to the resistance gene activity. Two time points of inoculation were selected for the analyses. At the first time point investigated, 24 hours post inoculation (hpi), bibliographic data report that the leaf rust pathogen has established haustoria in the mesophyll cells and has started intercellular hyphal growth [13,14]; Brian J. Steffenson, personal communication]. At 4 days post inoculation (dpi), the number of cells with established haustoria is higher than at 24 hpi and hyphae completed their intercellular growth. Since infection conditions applied in the present work are basically the same as those previously reported [13,14], we assumed that in our experiments the differentiation of infection structures and colonization process followed the same timing as described above. In agreement with previous observations, no disease symptoms were observed in both the NILs at 24 hpi, while at 4 dpi few chlorotic areas were observed in the susceptible NIL Bowman only (Figure 1A). In order to assess the success of the inoculation process, single plants of each biological replicate were left until 8 dpi. At this stage sporulating colonies were observed in Bowman while Bowman-*Rph15* exhibited only few chlorotic areas. To further verify that defence responses were properly deployed in the infected leaves used for proteomic analyses, quantitative RT-PCRs were conducted on genes encoding for oxalate oxidase, an H_2_O_2_ generating enzyme [15], and callose synthase, an enzyme involved in cell wall reinforcement [16]. For both genes expression was significantly increased by inoculation at 1 and 4 dpi (P<0.05, Methods) and was unresponsive by 8 dpi, with higher transcription level in Bowman compared to Bowman-*Rph15* (Figure 1B). These results demonstrate that active defence responses were triggered in both the genotypes at the inoculation time points used to detect leaf rust infection-dependent changes in protein accumulation.

Proteins obtained from the two infection time points were subjected to the proteomic analysis and three technical replicates were performed for each of two biological replicates. In the 2-DE maps, an average of 850 protein spots were visualized with the SYPRO Ruby fluorescent staining. After image and statistical analyses, no significantly differentially accumulated proteins were identified at 24 hpi in both genotypes (data not shown). In agreement with our results, no changes in protein accumulation were also detected at 3 dpi in the interaction between the leaf rust pathogen *P. triticina* and a susceptible wheat line [12]. Nevertheless, it is important to observe that at 18 hpi more than 1000 genes were differentially expressed in barley leaves of genotypes carrying QTLs for partial resistance to *P. hordei* in accordance with an active defence response at this infection time [10,11]. These findings underline, as expected, that a longer time is required to detect the response at protein level. Moreover, because of the proteomic approach adopted, only a part of the proteome was investigated in this study and this do not permit a satisfactory comparison between transcriptomic and proteomic data.

**Figure 2:**
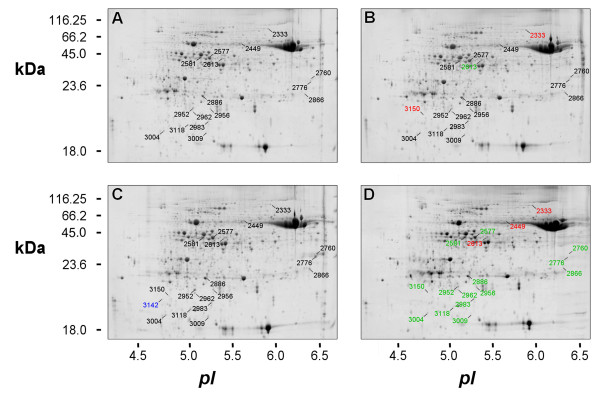
**2-DE maps.** Representative 2-DE maps of soluble protein fractions extracted from Bowman and Bowman-*Rph15* leaves at 4 days after mock inoculation (**A** and **C**, respectively) or after inoculation with leaf rust spores (**B** and **D**, respectively). Proteins (300 μg) were analyzed by IEF at pH 4–7, followed by 12.5% SDS-PAGE and visualized by SYPRO-staining. Numbers, corresponding to those in Table 1 and Figure 3, indicate the spots, identified by LC-ESI-MS/MS, showing significant changes of at least two-fold in their relative volumes (t-test, p < 0.05) after 4 dpi. Proteins that increased or decreased after this treatment are reported in red or in green, respectively. Spot 3142 is highlighted in blue, indicating its absence in the Bowman-*Rph15* inoculated sample.

**Figure 3:**
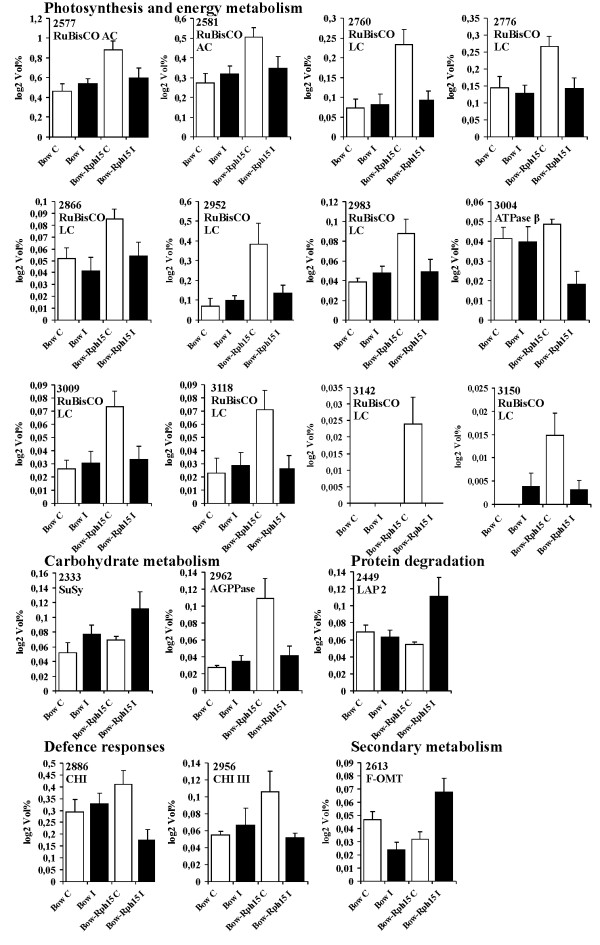
**Changes in protein accumulation in the two NILs in response to leaf rust infection.** Changes in the relative volumes of the identified proteins whose concentration is increased or decreased in the two NILs Bowman (Bow) and Bowman-*Rph15* (Bow-*Rph15*) at 4 days after mock inoculation (C) or after inoculation with leaf rust spores (I). Values are the means of six 2-DE gels derived from two independent biological replicates analyzed in triplicate (n=6). Error bars represent SD across all replicates. Numbers identify the spots as indicated in Table 1 and Figure 2; proteins were ordered into five functional classes, as indicated in Table 1.

Figure 2 reports 2-DE representative maps of proteins isolated from both the Bowman and the Bowman-*Rph15* in mock-inoculated and leaf rust inoculated conditions at 4 dpi. Eighteen protein spots showing significant differential accumulation in response to pathogen infection were identified at 4 dpi; differential accumulation was claimed only for spots in which normalized volumes of six replicates for each condition was showing an average fold change in their relative volumes of at least two folds (Table 1; Figures 2 and 3). In the susceptible NIL (Bowman) at 4 dpi only a few differences were detectable (Figure 2 A *vs* 2 B). In particular, two spots (2333 and 3150) were more abundant and one spot (2613) was less abundant in the rust inoculated sample with respect to the mock inoculated one. In the resistant NIL (Bowman*-Rph15*) a total of 15 spots were significantly down accumulated or absent (spot 3142) in the inoculated samples with respect to its control, while the levels of three protein spots rose in the inoculated sample with respect to the control condition (Figure 2 C *vs* 2 D).

**Table 1:** **List of the 18 spots identified by LC**-**ESI**-**MS**/**MS whose concentration is modulated by leaf rust infection in barley leaves**

***Spot ID***	***Accession number***	**Species**	***Protein description***	***EC***	***Abb****.*	***M***_***r***_^**a**^*/****pI***^**a**^	***M***_***r***_^b^*/ pI*^b^	Cov. (%) ^***c***^
**Photosynthesis and energy metabolism**								
**2577**	**Q40073**	*Hordeum vulgare*	**Ribulose bisphosphate carboxylase**/**oxygenase activase A**, **chloroplastic**		RuACS (b)	40.2 / 5.3	46.1 / 5.6 ^e^	43.5 ^e^
**2581**	**Q40073**	*Hordeum vulgare*	**Ribulose bisphosphate carboxylase**/**oxygenase activase A**, **chloroplastic**		RuACS (c)	40.8 / 5.2	46.1 / 5.6 ^e^	34.6 ^e^
**2760**	**P05698**	*Hordeum vulgare*	**Ribulose bisphosphate carboxylase large chain**	4.1.1.39	RuLC	25.4 / 6.4	52.9 / 6.2 ^e^	16.3 ^e^
**2776**	**P05698**	*Hordeum vulgare*	**Ribulose bisphosphate carboxylase large chain**	4.1.1.39	RuLC	24.2 / 6.4	52.9 / 6.2 ^e^	22.6 ^e^
**2866**	**P05698**	*Hordeum vulgare*	**Ribulose bisphosphate carboxylase large chain**	4.1.1.39	RuLC	22.3 / 5.2	52.9 / 6.2 ^e^	8.0 ^e^
**2952**	**P05698**	*Hordeum vulgare*	**Ribulose bisphosphate carboxylase large chain**	4.1.1.39	RuLC	21.3 / 5.1	52.9 / 6.2 ^e^	9.0 ^e^
**2983**	**P05698**	*Hordeum vulgare*	**Ribulose bisphosphate carboxylase large chain**	4.1.1.39	RuLC	20.1 / 5.3	52.9 / 6.2 ^e^	4.6 ^e^
**3004**	**XP**_**002465461**	*Sorghum bicolor*	**ATP synthase β chain**^**d**^		ATPase β	19.4 / 4.1	22.8 / 5.3	6.1
**3009**	**P05698**	*Hordeum vulgare*	**Ribulose bisphosphate carboxylase large chain**	4.1.1.39	RuLC	19.2 / 5.2	52.9 / 6.2 ^e^	4.2 ^e^
**3118**	**P05698**	*Hordeum vulgare*	**Ribulose bisphosphate carboxylase large chain**	4.1.1.39	RuLC	19.7 / 5.0	52.9 / 6.2 ^e^	6.9 ^e^
**3142**	**P05698**	*Hordeum vulgare*	**Ribulose bisphosphate carboxylase large chain**	4.1.1.39	RuLC	20.4 / 4.7	52.9 / 6.2 ^e^	4.4 ^e^
**3150**	**P05698**	*Hordeum vulgare*	**Ribulose bisphosphate carboxylase large chain**	4.1.1.39	RuLC	20.7 / 4.8	52.9 / 6.2 ^e^	4.8 ^e^
**Carbohydrate metabolism**								
**2333**	**CAZ64535**	*Hordeum vulgare*	**Sucrose synthase**	2.4.1.13	SuSy	88.2 /5.9	92.2 / 5.8	18.4
**2962**	**CAC32847**	*Hordeum vulgare*	**Adenosine diphosphate glucose pyrophosphatase**		AGPPase	21.0 / 5.1	19.5 / 5.7 ^e^	17.4 ^e^
**Protein degradation**								
**2449**	**XP**_**002454700**	*Sorghum bicolor*	**Leucine aminopeptidase 2,chloroplastic**^**d**^	3.4.11.1	LAP 2	57.0 / 5.6	61.8 / 7.6	20.0
**Defence responses**								
**2886**	**CAA55345**	*Hordeum vulgare*	**chitinase**	3.2.1.14	CHI	22.3 / 5.2	26.6 / 6.1	29.4
**2956**	**BAD31057**	*Oryza sativa*	**Chitinase III**-**like protein**		CHI III	21.2 / 5.3	18.9 / 6.5	12.7
**Secondary metabolism**								
**2613**	**CAA54616**	*Hordeum vulgare*	**Flavonoid 7**-**O**-**methyltransferase**	2.1.1.6	F-OMT	39.2 / 5.4	42.3 / 5.4	29.5

Statistical information about LC-ESI-MS/MS analysis are reported in Additional files 1 and 2.

Abb.: abbreviation.

a: experimental molecular weight and p*I*.

b: theoretical molecular weight and p*I*.

c: amino acid coverage (%).

d: annotation obtained by BLAST-P alignment analysis against the *Viridiplantae* subset of the nr-database at NCBI.

e: value referred to the mature form of the protein.

### Identification of Rph15-resistance gene-related proteins

The 18 spots (Table 1 and numbers in Figures 2 and 3) of interest were characterized by LC-ESI-MS/MS. The differentially modulated proteins could be grouped into the following five different functional classes: photosynthesis and energy metabolism, carbohydrate metabolism, protein biosynthesis and degradation, defence responses and secondary metabolism.

#### Photosynthesis and energy metabolism

Twelve of the identified proteins corresponded to photosynthetic or chloroplast-related proteins, including RuBisCo large chain (RuLC) and RuBisCo activase, while one spot represented an ATP synthase protein (spot number 3004).

**Figure 4:**
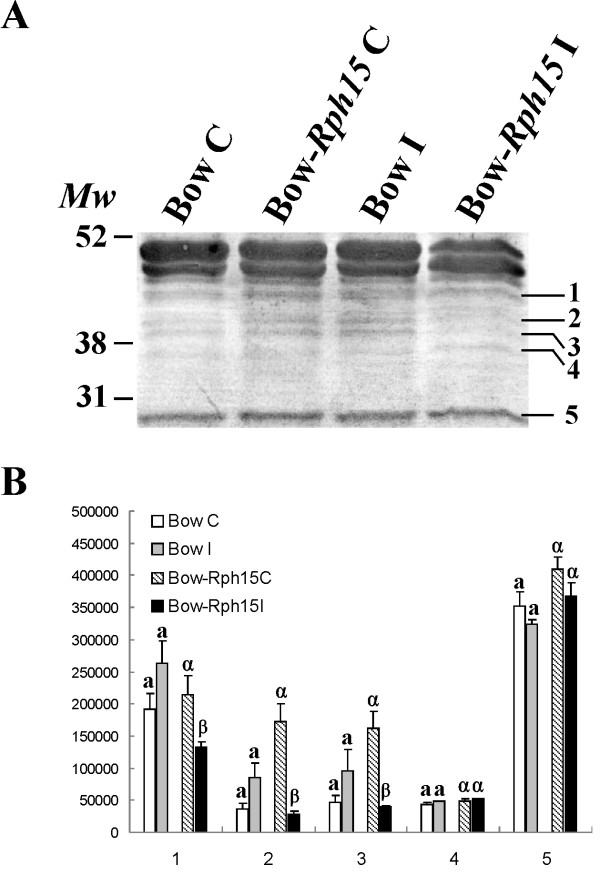
**Western blot analysis with α-Rubisco antibody.** (**A**) Western blot of five micrograms of proteins extracted from the two biological replicates of Bowman (Bow) and Bowman-*Rph15* (Bow-*Rph15*) at 4 days after mock inoculation (C) or after inoculation with leaf rust spores (I) were separated on 12% SDS-PAGE and hybridized with a Rubisco antibody. Bands representing the main Rubisco degradation forms are numbered from 1 to 5 on the Western right side. (**B**) Quantification data of Rubisco degradation forms; evaluation of degradation forms was conducted for the five bands indicated in (**A**) in Bowman (Bow) and Bowman-*Rph15* (Bow-*Rph15*) under control (C) or after inoculation with leaf rust spores (I) conditions. The data are the mean of three independent experiments. Different letters on the bars indicate significant difference P-values (P < 0.05) of the t-TESTs performed comparing control and inoculated samples.

RuBisCo is the most expressed protein in leaves, with about 30% of total leaf proteins [17,18] and an increase of RuBisCO degraded forms was generally observed in wheat leaves after *Fusarium graminearum* infection [19]. Many spots were identified in the present work as degradation products of RuLC; the experimental molecular weights were significantly lower than their theoretical values (Table 1), thus indicating a degradation of RuLC in the samples analyzed [20,21]. Degradation of photosynthetic apparatus may contribute to restrict pathogen growth into barley cells by promoting the activation of defences in non-infected cells because small peptide and amino acids derived from degradation can be directed to other metabolic pathways involved in defence [22,23]. We observed higher levels of RuLC fragments in the control samples than in the inoculated tissues of Bowman*-Rph15*, supporting that turnover of RuLC was markedly reduced in this genotype after infection. Interestingly, no statistically significant differences in RuLC fragments accumulation were observed between control and inoculated conditions in Bowman. To confirm the proteomics data, a Western blot analysis using an anti RuLC antibody was performed on the protein extracts of the two NILs under control and inoculated conditions (Figure 4). Results from this experiment support a reduction of RuLC degraded forms (indicated as numbers 1 to 5 in Figure 4A) occurring on infected Bowman-*Rph15* with respect to control tissues. In Bowman-*Rph15* inoculated sample was in fact evaluated a reduction of 38.2%, 84.1%, 75.28% and 10.12% of the RuLC degraded forms with respect to the control sample respectively for the major bands of the degradation forms numbered from 1 to 5 (Figure 4B). A similar RuLC degradation trend was observed in rice as induced by bacterial blight infection, in lesion mimic mutants [24-26] and in wheat after *Fusarium graminearum* infection [19]. Taken together, our results support the conclusion that in the resistant genotype Bowman-*Rph15* the Rubisco integrity was more preserved after infection with respect to control sample.

RuBisCo activase (RuACS) regulates RuBisCo activity by hydrolysing ATP to promote the dissociation of inhibitory sugar phosphates [27]. RuACS accumulation is reduced in the Bowman-*Rph15* NIL infected sample (protein spots numbers 2577, 2581) (Figure 3). In the susceptible NIL Bowman, no substantial alterations of RuLC and RuACS were observed. It can be hypothesized that this effect on RuACS is linked to a different strategy to modulate the phothosynthesis in Bowman-*Rph15*, the genotype in which the pathogen negatively affect RuBisCo degradation.

Spot 3004 represents an ATP-synthase β chain. This protein is involved in photosynthesis and oxidative respiration to drive synthesis of ATP in chloroplasts and mitochondria [28]. Accumulation of this protein was down-regulated after infection in the resistant NIL only (Figure 3). A decrease of proteins related to carbon metabolism and photosynthesis that include RuLC, RuACS and ATP synthase has been also observed in orange (*Citrus sinensis*) leaves after infection with the biotrophic bacterial pathogen *Xanthomonas citri* pv. *citri*[29]. Similarly, a down regulation of ATP-synthase subunits was observed in grape (*Vitis vinifera*) response to Flavescence dorée infection [30]. Similar observations were obtained at the transcriptomic level for barley leaves infected with the biotrophic powdery mildew fungus [31] and after an extensive analysis of the effects on photosynthetic genes of several biotic stresses in eight plant species [32]. These investigations suggest that a slow turnover of many photosynthetic proteins represent an adaptive consequence resulting in reduced energy supply when plants are facing a biotic attack and require to re-direct resources in immediate defence needs [27,32,33]. The reduced accumulation of these three proteins in the resistant NIL is therefore possibly consistent with a re-direction of the metabolism to fuel defence responses. Nevertheless, down accumulation of RuLC, RuACS and ATP synthase proteins was also observed in the leaves of rice *spotted leaf 6* mutants that undergo to spontaneous programmed cell death (PCD), caused by oxidative burst and membrane damage, in the absence of pathogen infection [18]. Since the *Rph15* resistance most likely involves an HR-dependent PCD response, similarly to that of most *Rph* genes conferring complete resistance [6], it cannot be excluded that the down-regulation of photosynthetic proteins observed in the resistant NIL could also depend from cellular damage caused by the HR response.

An higher accumulation of RuLC degraded forms was observed for Bowman-*Rph15* with respect to Bowman in the control samples (Figure 3). Also for RuACS and AGPPase, higher accumulation was present in Bowman-*Rph15* than in Bowman control samples. Conversely, this behaviour was not observed for several other protein spots (i.e. Susy, LAP2, OMT, ATPaseβ, see below), indicating a process specific for proteins involved in the photosynthetic apparatus. Since data are deriving from six replicates for each experiment (with two biological replicates) and SD detected for these proteins was low, it can be postulated that the observed trend represents a genuine differential accumulation among Bowman and Bowman-*Rph15* control samples ascribable to an unknown alteration of the cellular homeostasis orchestrated by the *Rph15* resistance gene or by another tightly associated sequence.

#### Carbohydrate metabolism

Two infection-modulated proteins identified in this study are related to sugar metabolism: sucrose synthase (SuSy, EC. 2.4.1.13) and adenosine diphosphate glucose pyrophosphatase (AGPPase). SuSy accumulation was significantly increased in infected leaves of the resistant NIL (spot 2333 in Table 1; Figures 2 and 3). SuSy catalyzes the reversible reaction from sucrose to fructose and UDP-glucose and an increase in this activity usually results in decreased starch biosynthesis. In addition, SuSy is the key enzyme of symplastic sucrose unloading and in concert with invertases, it can modulate the sink capacity of plant tissues [34]. SuSy has a dual role in producing both cytosolic ADPG, directed to starch biosynthesis, and UDPG, necessary for cell wall material and glycoprotein biosynthesis [35]. Defense-related SuSy activity may serve to allocate sucrose into callose deposition and other carbohydrate-consuming defense reactions, as supported by the observation of an increased SuSy activity in the resistant response of tobacco plants to *Phytophthora nicotianae*[36] and in several other plant–pathogen interactions (reviewed in [37]). A leaf rust infection-dependent induction of a callose synthase gene was observed in the present work (Figure 1B), suggesting re-directioning of sugars to structural components of the cell wall to constitute physical barriers to the infection.

A consistent decrease in ADP-glucose pyrophosphatase (AGPPase) abundance (spot 2962 in Figures 2 and 3) was observed in infected tissues of the resistant genotype only. AGPPase catalyzes ADP-glucose breakdown to produce AMP and G1P, and its reduced accumulation/activity drastically lead to a reduction in starch biosynthesis [38,39].

There are increasing evidences that the availability of soluble carbohydrates is a major factor in determining plant resistance to infections. The results of plant-pathogen interactions also depend on the rapid mobilization of carbohydrates and on the reprogramming of the carbon flow from sucrose to hexoses [36]. The data about SuSy and AGPPase suggest that in the resistant genotype under infection there could be a redirection of primary metabolism that leads to a reduction of starch biosynthesis in order to provide a strong supply to the hexoses pool. To further verify the role of sugar metabolism in *Rph15*-mediated response to leaf rust, an evaluation of reducing sugars and sucrose was conducted on control and inoculated conditions in both NILs (Bowman and Bowman-*Rph15*) (Table 2). While sucrose content was not affected by infection, reducing sugars were slightly but significantly increased (of about 12%) by inoculation in the resistant NIL and significantly decreased in the susceptible one. The reducing sugars increase supports a re-direction of the hexoses pool to defence pathways activated in the resistant NIL in response to the leaf rust pathogen infection.

**Table 2:** Levels of reducing sugars and sucrose in the leaves at 4 dpi or after mock inoculation

		**Reducing sugars**	**Sucrose**
		**(μmol g**^**-1**^**fr.wt)**	**(mg g**^**-1**^**fr.wt)**
**NILs Bowman**	**Control**	**6**.**39**^**a**^	**30**.**15**^**a**^
		± 0.19	± 2.66
	**Inoculated**	**5**.**87**^**b**^	**29**.**52**^**a**^
		± 0.09	± 2.58
**Bowman**-***Rph15***	**Control**	**6**.**09**^α^	**29**.**02**^α^
		± 0.29	± 2.79
	**Inoculated**	**6**.**85**^β^	**29**.**89**^α^
		± 0.25	± 0.55

The data are the means ± SE of three experiments run in triplicate (n = 9). Different letters on the values indicate significant difference *P*-values (P < 0.05) of the t-TESTs performed comparing control and inoculated samples.

#### Protein degradation

Protein spot number 2449 (Table 1; Figures 2 and 3) was characterized as a leucine aminopeptidase (LAP2) protein. LAP2 accumulation was observed in the inoculated sample of the resistant NIL only. Aminopeptidases represent plant responses to wound and pathogen stresses [40,41] and their activity (in association to other peptidases) is involved in the turnover of unfolded or damaged proteins that accumulate as a result of the oxidative burst. Furthermore, the role of aminopeptidases in the genesis of bioactive peptides in animals [42] and, more recently, in plants [43] highlighted their possible contribution in defence responses signalling. The modulation of the levels of LAP2 observed was *Rph15* resistance gene-dependent, consequently this protein could have a role both at the level of protein turnover, since the *Rph15* resistance response would most likely involve an HR-associated oxidative burst, and/or in resistance gene signalling. The fact that LAP2 is more abundant in Bowman*-Rph15* line under infection reinforces the idea that protein degradation could be one of the main mechanisms contributing to resistance deployment.

#### Defence responses

Two protein spots (2886 and 2956, Table 1, Figures 2 and 3) were respectively identified as chitinase and chitinase III-like. Protein abundance significantly decreased in the inoculated samples of the resistant NIL only at 4 dpi, while in the susceptible NIL the levels of chitinases were not affected. For both chitinases, the level of accumulated proteins was however constitutively higher in mock-inoculated tissues of the resistant NIL with respect to the susceptible one. This behaviour supports the possibility that a higher constitutive level of these defence proteins is associated with resistance and it operates as an early defence barriers against the pathogen infection. Similar results were found in the interaction between wheat and *Septoria tritici* where a higher chitinase activity, measured on whole leaf extracts, was observed in control with respect to inoculated plants during the first 7 dpi but the enzymatic activity did not correlate well with resistance in the host or the infection course of the pathogen [44]. Plant chitinases, however, are classified into seven classes (I-VII), on the basis of their structure, substrate specificity, mechanisms of catalysis, and sensitivity to inhibitors, with several members for each class [45,46]. Therefore it cannot be excluded that in the present work only two specific members of this class of defence proteins with peculiar roles in defence were detected.

#### Secondary metabolism

Spot 2613 was identified as a flavonoid 7-*O*-methyltransferase and the production of this protein was enhanced in infected leaves of the resistant NIL while in the susceptible NIL infection resulted in a significant down accumulation of the protein (Figures 2 and 3). This enzyme, that accumulated faster in incompatible interactions between barley and powdery mildew with respect to compatible interactions, was demonstrated to preferentially catalyze the methylation of the flavone apigenin, most likely leading to the production of phytoalexins [47]. Since methylated flavonoids have been found to have more antifungal activity than their unmethylated precursors [48,49], it is plausible that the protein accumulation in Bowman-*Rph15* represents a resistance gene-mediated defence response coupled to pathogen recognition, while in the susceptible NIL a successful leaf rust colonization is associated to the suppression of defence responses leading to reduction of 7-*O*-methyltransferase protein. Several fungal pathogens demonstrate to secrete protein effectors capable in suppressing resistance gene-based and basal defence responses [50-53]; it is therefore possible that *P. hordei* secreted effectors contribute to pathogen proliferation and disease development during the interaction with the susceptible NIL.

### Transcript analysis and correlation with proteomic data

**Figure 5:**
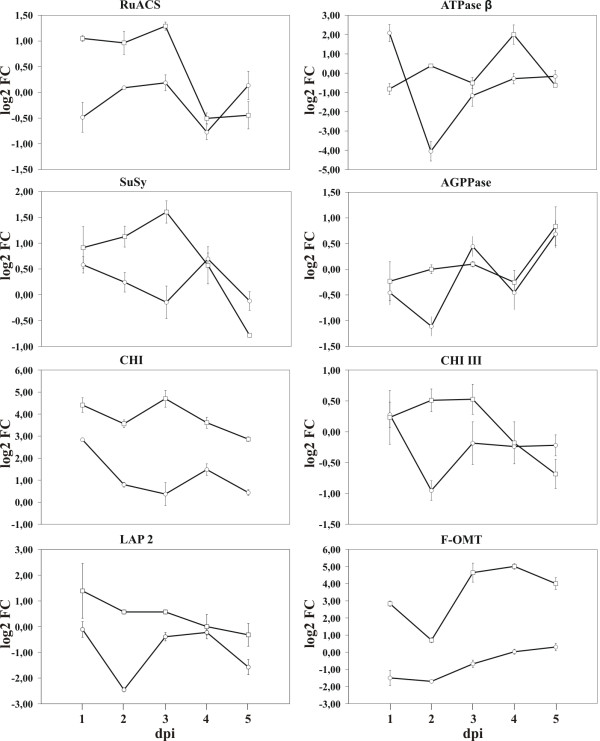
**Gene expression analysis of eight target genes.** Quantitative RT-PCR at 1, 2, 3, 4 and 5 dpi for eight genes in leaves of Bowman (open circles) and Bowman-*Rph15* (open squares). Values are expressed as log2 fold changes of transcript levels in the inoculated samples with respect to the transcript levels in mock-inoculated barley leaves. Numbers and name abbreviations are corresponding to those in Table 1. Error bars represent SD across all RT-PCR replicates (three from each of two independent inoculations). Statistical significance of differential expression for the tested genes was evaluated with a Wilcoxon two group test (P<0.05, Methods).

Eight genes involved in protein accumulation changes were evaluated at the transcription level by quantitative RT-PCR (qRT-PCR) using RNAs obtained from inoculation time course experiments that included also the inoculations time points used for proteomics analyses (Figure 5).

No or weak correlations between mRNA and protein levels were observed for the LAP2, ATP synthase and SuSy. For the last gene, only a weak correlation with the proteomic data was observed in Bowman-*Rph15* being SuSy transcription up regulated until 3 dpi but then down regulated. This transcriptional response is coherent with both the observed increase of Susy protein (Figure 3) and reducing sugars (Table 2) at 4 dpi. In Bowman, SuSy gene expression increased from 3 to 4 dpi suggesting a delayed plant response to the fungus. Nevertheless, bibliographic data indicate that pathogen responsiveness of SuSy transcription is highly variable ranging from down regulation during a barley-*B. graminis* f. sp. *tritici* non-host interaction [54] to enhanced transcription observed in Arabidopsis plants infected with *Plasmodiophora brassicae*[55] and in phytoplasma-infected grape plants [56].

In agreement with the proteomic data, expression of RuACS and AGPPase genes decreased at 3 to 4 dpi, while transcript levels of the two chitinase genes tested decreased in the resistant NIL starting from 3 dpi. Proteomics and transcription analyses both suggested an up-regulation of flavonoid 7-O-methyltransferase in Bowman-*Rph15* after leaf rust infection while the gene transcription was repressed by pathogen infection in the susceptible NIL. An up-regulation for the flavonoid 7-O-methyltransferase gene was observed also during a transcript profiling study of the broad spectrum race-nonspecific leaf rust resistance gene *Lr34* after interaction with *P. triticina*, but not in the compatible interaction or in the race-specific resistance gene *Lr1*[5], thus supporting a possible important role of this enzyme in defence responses involving lignin biosynthesis and production of phytoalexins antimicrobial compounds [57].

In our experiments we observed that, for the time points subjected to parallel proteomics and transcriptomic analyses, 62,5% of the protein changes correlate with the transcriptomic data, since for five genes out of 8 analyzed, an agreement was observed between protein accumulation and transcriptional change at the corresponding time points. Higher level on incongruent expression between mRNAs and proteins was however frequently observed by other groups, in other species and experimental conditions [58-62] and is most likely a result of the biology of gene expression which includes various levels of regulation during protein synthesis: post-transcriptional, translational, and post-translational. Thus, integrated analysis of both mRNAs and proteins is crucial to gain further insights into complex biological systems.

## Conclusions

In this work, proteomics and a complementary transcriptomic approaches were applied to two NILs differing for the presence of the highly effective broad spectrum *Rph15* leaf rust resistance gene. The genetic materials analysed allows to relate the observed responses to the presence/absence of the resistance gene. We observed that the susceptible NIL was basically unresponsive to pathogen infection at the time points analyzed, indicating that basal defences are suppressed and/or delayed by pathogen effectors or by the lacking of effective signalling pathways. In a study on the susceptible interaction between the leaf rust pathogen *P. triticina* and wheat, changes in proteome were identified only after 6 dpi [12], indicating that a similar delay in defence towards leaf rusts could represent a common feature of susceptible genotypes. In Bowman, a substantial transcripts down regulation in infected tissues, with respect to control samples, was observed for SuSy, LAP2 and chitinases, while down regulation of both, protein and transcripts, was observed for flavonoid 7-O-methyltransferase, supporting a suppression of defence responses by pathogen effectors. In the resistant genotype we observed a reduction of RuLC degradation products together with a down regulation of RuACS and ATP synthase. These responses could be associated to the re-direction of the metabolism to sustain defence responses and, possibly, to cellular damage caused by HR responses. Protein changes for Bowman-*Rph15* also supports a i) carbohydrate metabolism variation leading to a reduction in starch biosynthesis possibly connected to allocation of sugars into carbohydrate-consuming defence reactions (this hypothesis was confirmed by the quantification of reducing sugars), ii) an increase of proteins related to proteolitic activities and iii) accumulation of enzymatic proteins involved in phytoalexin and lignin production. These responses were observed in the resistant NIL only and are therefore dependent from the resistance pathways activated, upon pathogen recognition, by the *Rph15*-mediated resistance signalling. Since none of these changes were highlighted at 24 hpi, it can be assumed that accumulation of defence-related proteins starts in the interval between 24 hpi and 4 dpi. In conclusion, the proteomic approach adopted, complemented with transcriptomic analysis, provided a picture about the timing, entity and the nature of differential defence responses in basal resistance and *Rph15*-gene mediated resistance.

## Methods

### Fungal and plant materials

The study was performed with barley leaf rust causal agent *Puccinia hordei* isolate 4, kindly provided by Prof. Brian J. Steffenson (Department of Plant Pathology, University of Minnesota, USA). The fungus urediniospores were propagated on the susceptible barley cv. Bowman and stored at −80°C. Inoculation was carried out after spores activation by short thermal shock at 37°C. Active spores were implemented on Bowman barley leaves and then collected to infect barley seedlings.

Two barley (*Hordeum vulgare*) lines, near-isogenic (NILs) for the leaf rust resistance gene *Rph15*, were used in this study. The cv. Bowman is susceptible to the leaf rust isolate 4 while the line Bowman-*Rph15* is resistant. These barley genotypes were kindly provided by Prof. Jerry Franckowiak (North Dakota State University, USA). All barley seedlings were grown at 16 h (21°C) in the light and 8 h (16°C) in the dark in growth chamber. Barley seedlings at the first-leaf stage were inoculated with the spores of the leaf rust isolate 4. Rust spores were mixed with talcum powder (1:10 v/v) and used for barley leaves infection at a density of about 200 spores per cm^2^. Mock inoculation of the two NILs was carried out with talcum powder only. Seedlings were transferred for 24h at 20°C in complete darkness and 100% humidity, and then placed at 20°C with 14 h in the light and 10 h in the dark with 60% humidity. Two biological replicates were performed for each experiment. At the end of the infection time points, leaf samples were collected and stored at −80°C.

### Protein extraction

Barley leaf tissues were transferred into a pre-chilled mortar where they were ground to a fine powder with a pestle in liquid nitrogen and homogenized in extraction buffer (0.5 M Tris–HCl pH 8.0, 0.7 M sucrose, 10 mM sodium fluoride, 1 mM PMSF, 0.1 mg mL^-1^ Pefabloc, 0.2% (v/v) Triton X-100, 2 μL mL^-1^ Phosphatase Inhibitor Cocktail (Sigma), 0.2 (v/v) β- mercaptoethanol) and PVPP 1% on ice. Phenol extraction method of proteins was according to [63] with few modifications. Samples were centrifuged at 4500 g at 4°C for 40 min and the pellet was dried. The pellet was solubilized in isoelectrofocusing buffer (7 M Urea, 2 M Thiourea, 3% w/v CHAPS, 1% w/v NP40, 50 mM DTT, 2% v/v ampholytes), incubated at room temperature for 1 h and centrifugated at 10000 rpm for 10 minutes at room temperature. Supernatant was collected and stored at −80°C until further use. Protein concentration was determined by 2D Quant Kit (GE Healthcare) and Bradford method, according to manufacturer’s instructions (Biorad protein assay kit), using BSA as the standard. For each sample two different extractions were performed.

### Two-dimensional electrophoresis

Protein samples (300μg) were loaded on pH 4–7, 24 cm IPG strips (GE Healthcare Life Sciences, USA) passively rehydrated 10 hours in 7 M urea, 2 M thiourea, 3% (w/v) CHAPS, 1% (w/v) NP40, 10 mM DTT, 0.5% (v/v) ampholytes, traces of Orange G before use in reswelling tray under mineral oil. IEF was conducted at 20°C with current limit of 75μA/strip in a Ettan IPGphor III (GE Healthcare Life Sciences, USA) following the manufacturer’s protocol. After IEF, strips were equilibrated by gentle stirring for 15 minutes in an equilibration buffer (100 mM Tris–HCl pH 6.8, 7 M urea, 2 M thiourea, 30% (w/v) glycerol, 2% (w/v) SDS) added with 0.5% (w/v) DTT for disulfide bridges reduction, and for additional 15 minutes in the same equilibration buffer supplemented with 4.5% (w/v) iodoacetamide for cysteine alkylation and 0.002% (w/v) bromophenol blue. Second-dimensional SDS-PAGE [64] was run in 12.5% acrylamide gels using ETTAN DALT*six* apparatus (GE Healthcare Life Sciences, USA). Running was conducted for 30 min at 5 W/gel followed by 15 W/gel, until the bromophenol blue dye front left the gel. Three technical replicates were performed for each biological replicate. Experimental pI was determined using a 4–7 linear scale over the total length of the IPG strip. M_r_ values were calculated by mobility comparison with protein standard marker included in fluorescent staining (Molecular Probes, Inc.)

### Protein visualization and data analysis

Silver staining: after SDS-PAGE Tris-Tricine, gels were fixed in ethanol/acetic acid solution and stained with silver nitrate as previously described [65]. Coomassie staining: proteins were visualized using colloidal Coomassie Brilliant Blue G-250 (cCBB) procedure, as previously described [66]. Images of gels stained with silver and Coomassie were scanned with an EPSON ScanMaker i900 MicroTek Scanner. Fluorescent staining: as a fluorescent stain for total proteins, the SYPRO Ruby stain (Molecular Probes), with a sensitivity of 0.5-5 ng of protein [67], was used. After 2-DE, gels were fixed twice in 50% (v/v) methanol and 7% (v/v) acetic acid for 30 minutes. Each single gel was immersed overnight in SYPRO Ruby and then destained in 10% (v/v) methanol and 7% (v/v) acetic acid and finally washed in deionized water. Fluorescent gel images were acquired on Typhoon 9210 laser scanner (GE Healthcare) at 280 nm and 450 nm excitation and 610 nm bandpass emission filter. Spot detection, matching and image analysis were performed using ImageMaster 2D Platinum Software 6.0 (GE Healthcare Life Sciences, USA). Automatic matching was complemented by manual matching. Spot volumes were normalized and expressed as the percentage of the total volume of all the spots present in the gels (six replicate gels for each condition). During the analysis, only spots showing at least two-fold change in their relative volumes were considered for subsequent analyses. In order to find differentially expressed proteins, the values were log(z+1) transformed and subjected to a two-way ANOVA test to verify if their changes in expression were statistically significant (p<0.05), using genotypes and treatments as factors. The softwares Systat and STATISTICA were used for statistical analysis.

### Protein in-gel digestion and mass spectrometry protein characterization

Spots were excised from cCBB-stained 2-DE gels and in-gel digested with trypsin [Sequencing grade modified Trypsin V5111, Promega, Madison] as previously described [68]. The LC-ESI-MS/MS experiments were conducted using a Surveyor (MS pump Plus) HPLC system directly connected to the Nano-ESI source of a Finnigan LCQ DECA XP MAX ion trap mass spectrometer (ThermoFisher Scientific Inc., Waltham, USA). Chromatography separations were carried out on a ZORBAX 300SB-C18 column (75 μm I.D × 150 mm length, 3.5 μm particle size, Agilent Tecnologies ®), using a linear gradient from 5 to 60% solvent B [solvent A: 0.1% (v/v) formic acid; solvent B: ACN containing 0.1% (v/v) formic acid] with a flow of 300 nl/min. Nano-ESI was performed in positive ionization mode with spray voltage and capillary temperature set to 1.7 kV and 180°C, respectively. Data were collected in the full-scan and data dependent MS/MS mode with collision energy of 35% and a dynamic exclusion window of 3 min. Spectra were searched by TurboSEQUEST® incorporated in BioworksBrowser 3.2 software (ThermoFisher Scientific Inc., Waltham, USA) against the *Hordeum vulgare* protein subset (*7825 entries*) and against the *Hordeum vulgare* EST subset (*525775 entries*), both downloaded from the National Center for Biotechnology Information ( http://www.ncbi.nlm.nih.gov). The searches were carried out assuming parent ion and fragment ion mass tolerance of ± 2 Da and ± 1 Da, respectively, one possible missed cleavage *per* peptide, fixed carboxyamidomethylation of cysteine and variable methionine oxidation. Positive hits were filtered on the basis of peptides scores [Xcorr ≥ 1.5 (+1 charge state), ≥ 2.0 (+2 charge state), ≥ 2.5 (≥ 3 charge state), ΔCn ≥ 0.1, peptide probability < 1 × 10^-3^ and Sf ≥ 0.70] [69]. If needed, identified peptides were used in protein similarity search performed against the *Viridiplantae* subset of the NCBI-nr database using the FASTS algorithm [70]. Physical properties of the characterized proteins were predicted by *in silico* tools at ExPASy ( http://www.expasy.org) (Additional files 1 and 2).

### Western blot analysis

Samples (5 μg) were diluted with an equal volume of SDS-PAGE buffer [50 mM Tris–HCl (pH 6.8), 4% (w/v) SDS, 12% (w/v) glycerol, 2% (v/v) b-mercaptoethanol, and 0.01% (w/v) bromophenol blue] and heated for 5 min at 90°C, separated by SDS-PAGE using 10.0% acrylamide according to Laemmli (1970) and then electrophoretically transferred to a polyvinylidene difluoride (PVDF) filter using a semidry blotting system (NovaBlot, Pharmacia, Sweden) with a buffer containing 10 mM 3-cyclohexylamino-1-propanesulphonic acid (CAPS, pH 11 with NaOH) and 10% methanol. Filters were blocked for 1 h with TBS-T buffer [50 mM Tris–HCl (pH 7.6), 200 mM NaCl, and 0.1 % (v/v) Tween 20] supplemented with 3% (w/v) of albumine. The TBS-T buffer was used as an incubation medium throughout the procedure. Filters were incubated overnight at 4°C with primary polyclonal antibodies against Rubisco large subunit using a 1:10.000 dilution (Agrisera, AS03 037). After washing with TBS-T, the filters were incubated for a further 2 h at room temperature with a secondary antibody (alkaline-phosphatase-conjugated anti-rabbit immunoglobulin G). The blot was developed with nitroblue tetrazolium and 5-bromo-4-chloro-3-indolyl phosphate (FAST BCIP/NBT, Sigma). Three technical replicates were performed for the hybridization analyses. Quantification of the RuBisCO degraded forms was conducted by using the software Image Quant (Molecular Dynamic), version 5.2.

### RNA extraction and quantitative RT-PCR analysis

Eight target genes identified from proteomics analysis were selected for transcriptional analysis. Total RNA was extracted from 500 mg of frozen leaves powder of mock-inoculated and *P. hordei-*inoculated plants using TRI reagent® solution (Applied Biosystems, Foster City, CA, USA), according to manufacturer’s instruction. The RNA pellet was dissolved in 100 μl of autoclaved DEPC water and then stored at −80°C. RNA was extracted in a time course experiments from 24 hours to 5 days after pathogen inoculation. Nucleic acid quality was estimated using the Bioanalyzer software (Agilent Technologies, Santa Clara, CA, USA), while RNA concentrations were measured with a spectrophotometer (Beckman Coulter, CA, USA) and only RNA samples with an A_260_/A_280_ ratio in the range 1.8-2.0 were used in RT-PCR analysis. Sequences of the primers and reaction conditions used for RT-PCRs were as previously described [71]. Quantitative RT-PCR was performed using a One Step Real Time PCR System (Applied Biosystems, Foster City, CA, USA) and 100 ng of total RNA *per* reaction. Primers were designed using Primer3 software ( http://frodo.wi.mit.edu/primer3/) (Table 3) and primers specificity was evaluated by blasting primer sequences against the NCBI database. The barley β-actin constitutively expressed gene was used as reference gene for normalization. Standard variation in all samples was lower than 10%. PCR amplifications were performed in 25 μl of final volumes containing 2x QuantiFast SYBR® Green Master Mix (Qiagen, Hiden, Germany) and including ROX™ as passive reference dye, 400 μM each primer and 0.25 U/μl Multiscribe™ Reverse Transcriptase (Applied Biosystems). Three technical replicates for each of the two biological replicates were performed. Relative gene expression was calculated using the 2^-ΔΔCt^ method [72]. For all the genes tested by quantitative RT-PCR, a Wilcoxon two group test [73,74] was used to analyze the ΔCt values (Ct _target_-Ct _βact_) in infected and un-infected samples at each time point of inoculation. Data from two biological replicates with three technical replicates each were used for the analysis. In all the time points where an increased or decreased transcription of the genes in response to pathogen inoculation was observed, the test yielded *P*-values <0.05, indicating that ΔΔCt was significantly different from 0 and that there was a significant effect. No statistically different ΔΔCt values were observed for samples in which no transcriptional variations were detected by quantitative RT-PCR.

**Table 3:** **List of the genes whose transcription profile was evaluated by qRT**-**PCR**

**Gene name**	**Accession number**	**Primer**	**5**^′^-**3**^′^**primer sequences**
Oxalate oxidase	Y14203	F	CTGGCTGTTGAAGGACACAA
		R	TGACTCCGGAAACAAGCTCT
Callose synthase	AY177665	F	CATCAAGGAATCAGCTGCAA
		R	TCGCATGAACAAAGAGTTCG
Sucrose synthase	X65871	F	AAGCTGAAAGGCCATATCCGTT
		R	AGAATGCAGGCTGCACAAATG
Adenosine diphosphate glucose pyrophosphatase	AJ291451	F	TCATCAGCTCCTCCTCCAAC
		R	TGCCACCGTTGTACTGGTAG
Flavonoid 7-O-methyltransferase	X77467	F	CGAGGCTTTCCCTTATGTCA
		R	ACTCCATCATGTCACCAGCA
Leucine Aminopeptidase 2	AK248195	F	TCGGGCTCACCAAGGCCAACG
		R	GAGGATGTCGCCCTTCCAGTCG
Rubisco activase	M55447	F	TCCAAAAACTTCATGACCCTGC
		R	CGAACACAAGCTCACACTGGAA
ATP synthase B chain	EU963772	F	AGAAGGCCGCACTGTTTGACT
		R	CATCCATGAACTTGCCTAGCG
Chitinase	X78672	F	GCCACGTCTCCACCCTACTA
		R	ACCGTTCTGAAGGACACCAC
Chitinase III	AK251032	F	TAAGCTGTGCCGACTGAATG
		R	CACTGCAAACCACAACATCC
Actin	AJ234400	F	ACCTCGCTGGGCGTGACCTAACTG
		R	TGGTCTATGGATTCCAGCAGCTTCC

For each gene the accession number and primer sequences are provided.

### Determination of reducing sugars and sucrose

Reducing sugars, sucrose and amino acids were extracted by homogenizing frozen tissues in 5 volumes of ice-cold 0.5 M perchloric acid (PCA). The homogenate was centrifuged for 20 min at 11,000 *g* at 4°C and the resulting pellet was washed with the same volume of PCA and then centrifuged again in the same conditions. KOH was added to the collected supernatant (to pH 7.6) to remove excess PCA. Reducing sugars were measured according to the colorimetric method by [75]. Total soluble sugars were determined by the same method boiling an aliquot of PCA extract for 1 h before neutralization. Sucrose was estimated from the difference between total soluble and reducing sugars.

## Abbreviations

NILs: Near isogenic lines; RuLC: Ribulose bisphosphate carboxylase large chain; RuACS: Ribulose bisphosphate carboxylase/oxygenase activase A chloroplastic; ATPase β: ATP synthase β chain; SuSy: Sucrose synthase; AGPPase: Adenosine diphosphate glucose pyrophosphatase; LAP 2: Leucine aminopeptidase 2 chloroplastic; CHI: Chitinase; CHI III: Chitinase III-like protein; F-OMT: Flavonoid 7-O-methyltransferase.

## Competing interests

The authors declare any competing financial or other interest in relation to this work.

## Authors’ contributions

GV, LE and LC planned and supervised the work. LB carried out protein extraction, 2-DE gel analysis, statistical analysis, transcriptional analysis and drafted the MS. BP carried out protein characterization by LC-ESI-MS/MS, analyzed the MS data. ASN helped to perform 2-DE gel analysis and carried out statistical analysis. BP, ASN and LE carried out sugar and Western blot analyses. All the authors contributed to the final version of the manuscript. All authors read and approved the final manuscript.

## Supplementary Material

Additional file 1 **Caption of Additional file**2**.** Click here for file

Additional file 2 **Data on protein identification by LC-ESI-MS/MS and bioinformatic analysis.** Table shows the sequence of the peptides identified by MS/MS and the statistical information related to peptides, proteins and alignment analyses. Click here for file
