# Supplementary material for: Proteomic characterization of the Rph15 barley resistance gene-mediated defence responses to leaf rust
Source: BMC Genomics. 2012 Nov 20;13:642. doi: 10.1186/1471-2164-13-642 (PMC3541957; doi:10.1186/1471-2164-13-642)
Supplement: Additional file 1 — Caption of Additional file2. [file 1471-2164-13-642-S1.doc]

**Caption of the Excel file named “mass spectrometry data (Hordeum 2009)”.**

**Data regarding LC-ESI-MS/MS and bioinformatics analyses.** The table shows the sequence of all the peptides identified by MS/MS and the associated statistical information obtained from database searches conducted by BioworksBrowser using TurboSEQUEST® software. For each identified protein, statistical information related to direct protein database search or to alignment analysis of identified peptides by FASTS software are reported. **Spot ID**: spot identifier number. **Protein A.N.**: protein NCBI accession number (version). **DB**: database downloaded from NCBI; NRH: subset of the *Hordeum vulgare* proteins (*7825 entries*); EST: subset of the *Hordeum vulgare* ESTs (*525775 entries*). **n. pep**.: number of unique peptides used to identify the protein. **a.a. cov. (%)**: sequence coverage %. **Sf (pro):** protein SEQUEST Sf score. **FASTS (*E*) value**: FASTS expectation (*E*) values of the entry resulting from the alignment of peptides against *Viridiplantae* subset of nr-NCBI database. **Protein / EST A.N.**: NCBI accession number (version) of the identified protein or ESTs. **Peptide**: sequence of the identified peptide; the symbol M* indicates oxidized methionine. **MH+**: molecular mass of the peptide; **z**: charge state of the peptide**. Sf (pep):** SEQUEST Sf score of the peptide. **Xcorr**: SEQUEST cross-correlation value. **ΔCn**: delta correlation value. **Sp**: SEQUEST preliminary score. **(a):** values referred to the mature form of the protein.
